# Sugar Treatment of Tuberculosis

**Published:** 1919-03

**Authors:** 


### Sugar Treatment of Tuberculosis.

Lieutenant Colonel Case, of the United States Army in France, says that the sugar treatment of tuberculosis announced by Dr. Lo Monaco of Rome, when tested in the treatment of patients in several French hospitals proved ineffective and of no value. This explodes another much vaunted theory. The best way to treat tuberculosis, now known to the medical profession, is to increase the vital resistance by properly building up the body, allowing the system to secure advantage of the natural healing processes.

